# Variations of intronic branchpoint motif: identification and functional implications in splicing and disease

**DOI:** 10.1038/s42003-023-05513-7

**Published:** 2023-11-10

**Authors:** Jiuyong Xie, Lili Wang, Ren-Jang Lin

**Affiliations:** 1 Department of Physiology & Pathophysiology, Max Rady College of Medicine, Rady Faculty of Health Sciences, University of Manitoba, Winnipeg, MB R3E 0J9 Canada; 2 Department of Systems Biology, Beckman Research Institute, City of Hope National Medical Center, Duarte, CA 91010 USA; 3 Center for RNA Biology & Therapeutics, Beckman Research Institute, City of Hope National Medical Center, Duarte, CA 91010 USA

**Keywords:** RNA splicing, Cancer genomics

## Abstract

The branchpoint (BP) motif is an essential intronic element for spliceosomal pre-mRNA splicing. In mammals, its sequence composition, distance to the downstream exon, and number of BPs per 3´ splice site are highly variable, unlike the GT/AG dinucleotides at the intron ends. These variations appear to provide evolutionary advantages for fostering alternative splicing, satisfying more diverse cellular contexts, and promoting resilience to genetic changes, thus contributing to an extra layer of complexity for gene regulation. Importantly, variants in the BP motif itself or in genes encoding BP-interacting factors cause human genetic diseases or cancers, highlighting the critical function of BP motif and the need to precisely identify functional BPs for faithful interpretation of their roles in splicing. In this perspective, we will succinctly summarize the major findings related to BP motif variations, discuss the relevant issues/challenges, and provide our insights.

## Abstract (web summary)

In this Perspective, motif variations and current insights into the branchpoint motif, an essential intronic element for spliceosomal pre-mRNA splicing, are discussed.

## Introduction

**Fig. 1:**
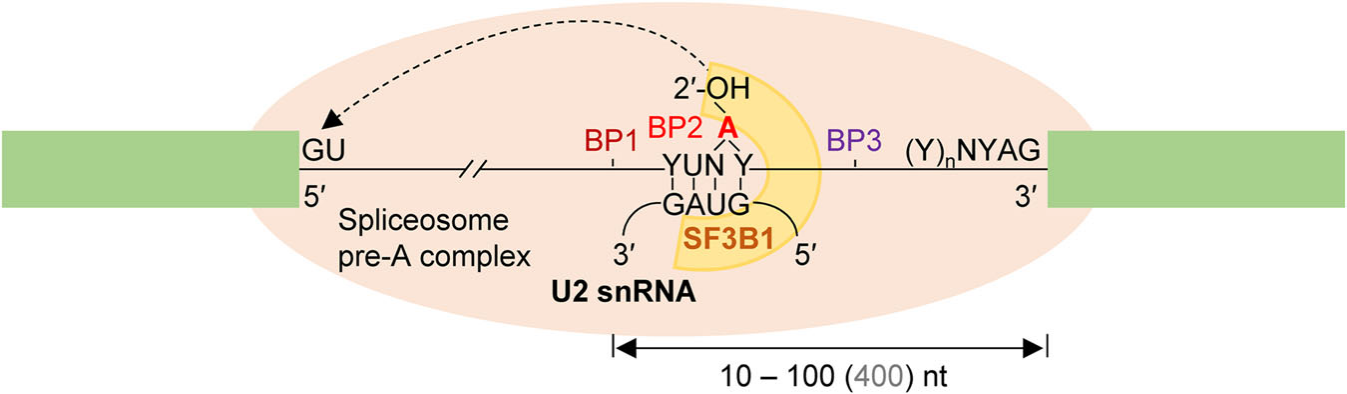
Diagram of the variations of the BP motifs in human introns. The BP location (up to 400nt) from the intron end and number of BPs (three BPs are shown as an example with details for the BP2 chosen for lariat formation here) per 3′ splice site are depicted. The SF3B1 (horseshoe) is shown in the pre-A complex (oval) before clamping on the U2-BP motif helix during PRP5-facilitated BP proofreading. Green boxes: exons.

Of the essential intronic sequences for splicing, the branchpoint (BP) motif perhaps has the most variations in terms of sequence composition, distance to the downstream exon, and the number of BPs per 3′ splice site (Fig. 1). Failure of proper BP recognition due to genetic variants of the motif itself or the *trans*-acting factors that recognize it causes human genetic diseases or cancer^1–3^. However, accurately predicting, validating, and interpreting the functional BP has been complicated by the variations of the BP motifs.

In spliceosomal RNA splicing, the BP is mostly an adenosine^1^, which is located largely within 10–60nt from the downstream exon^4,5^, with the peak position at about 25nt in humans^4–6^. Initially, it is bound by the branchpoint binding protein (BBP, also known as splicing factor 1, SF1), after the U1 snRNP binding the 5′ splice site and the U2AF35/65 (U2AF1/U2AF2) heterodimer to the 3′ AG and polypyrimidine tract^7^. Then, facilitated by the ATP-dependent DEAD box helicase UAP56/DDX39B^8^, BBP/SF1 is displaced by SF3B1 from the U2 snRNP^9^, recruited via their interactions with the U2AF65/U2AF2^8–10^. As this shift occurs, the U2 snRNA forms base pairs with the branchpoint motif, which is further stabilized as SF3B1 wraps around the BP region. The process is also coordinately regulated by the BP upstream anchoring site^11^, *cis*-acting splicing enhancers and silencers and *trans*-acting factors like serine/arginine-rich (SR) proteins and heterogeneous nuclear ribonucleoproteins (hnRNP)^12,13^. These stepwise processes collectively control the 3′ splice site recognition and regulate alternative pre-mRNA splicing.

The BP motif plays an essential role by attacking the first nucleotide of the intron (the 5′ guanine) to form a 2′, 5′-phosphodiester bond, resulting in a lariat intermediate^5,14,15^. For this to occur, the BP adenosine needs to be bulged from the helix formed by the BP motif and U2 snRNA. Yeast Hsh155 (SF3B1 in humans) interacts and eventually clamps the helix through its HEAT domain, converting the pre-A to the A complex during spliceosome assembly^16,17^. This transesterification reaction is conserved from yeast to humans but the BP motif sequence, location, and particularly the number of BPs per 3′ splice site may vary among different introns and species.

Here we will briefly summarize the variations of the BP motifs in biology and diseases, related issues in BP identification, and provide our views to address the issues in future BP studies.

## BP motif variations, advantages, diseases, and challenges

### BP motif variations

The variations of the BP motifs appear to increase on an evolutional scale. For instance, in fungi and protists, variation of the BP motif is relatively less than that in metazoans particularly humans^18^, as revealed by massive lariat RNA sequencing (RNA-Seq), iCLIP-Seq (individual-nucleotide resolution UV crosslinking and immunoprecipitation - RNA sequencing), and/or consensus motif prediction^1,4,5,14,18–20^. Unicellular yeasts have a consensus motif TACTAAC (underlined: BP adenosine), multicellular fungi Ascomycetes CT(G/A)AC, and humans YTNAY (Y: pyrimidine C or T; N: any nucleotide)^1,18,21^, with increasing sequence degeneration, although TACTAAC remains the preferred motif in humans^22^. In mammalian cells, the BP adenosine can be replaced by cytidine or guanosine in some introns^1,19,23^.

The locations of BP by its distance from the downstream exon may vary as well. The location is relatively fixed in some yeasts or protists such as *Y. lipolytica* and *B. microti strain RI* (six nucleotides upstream, A_-6_) and *E. invadens strain IP1* (eight nucleotides upstream, A_-8_)^18,24^. However, in other species, it is widely distributed within 10–100 nt upstream from the 3′ end of the intron^25^. The variations in humans can be even larger; for instance, 838 possible branchpoints were detected at up to 400nt away from their downstream exons^25^, with long U-rich polypyrimidine tracts and an AG-exclusion zone (AGEZ) ≥100 nt upstream from the 3′ AG. The distant BP and related 3′ splice site arrangement likely play a role in the mutually exclusive alternative splicing^25–27^.

The number of BPs per 3′ splice site may also exhibit variation. For example, the upstream BP of the alpha-tropomyosin exon 3 was mapped to A_-175_ by primer extension but its mutation abolished only 50% of exon 3 inclusion, suggesting that other BPs used for the same 3′ AG and downstream exon inclusion^25^. Indeed, A_-182_ together with A_-175_ mutation abolished all exon 3 inclusion^25^. In fact, multiple BPs that could result in the use of the same 3′ splice site seem to be more prevalent (>60%) for human introns as revealed by lariat RNA sequencing^1,19^.

Thus, the branchpoint motif has evolved from a relatively fixed sequence of TACTAAC in unicellular yeast to the divergent YTNAY in humans. The location of branchpoints varies from relatively fixed positions (A_-6_ and A_-8_) in some protists to a wide range of locations (A_-10_ to A_-400_) upstream of the intron end. Moreover, the number of BPs for a 3′ splice site has evolved from one in yeasts and protists to mostly more than one BP in humans.

Besides the above variations in the regular splicing that joins exons into mature RNA, a suboptimal BP consensus motif CTNA with more distant BPs (A_-29_) is found in the recursive splicing of zero-nucleotide exons in long introns, in contrast to the consensus CTAAT with A_-14–__-26_ in shorter introns in the *Drosophila* genome^28–30^.

### The advantages of BP motif variations

Despite the evolutionary changes of the BP motifs, the majority of the *U2* snRNA genes in fungi contain a GTAG motif (GΨAG in the U2 snRNA, Ψ: pseudouridine) that is complementary to the CTAAC BP sequence^18^. Here the BP adenosine, sandwiched between its flanking U2-pairing nucleotides but itself without a complementary base in the U2 snRNA, protrudes from the helix, facilitated by the pseudouridine^31^. In humans, about 65% of the 172 human *U2* snRNA sequences collected in the Rfam database (RF00004) also contain the GTAG motif within 50nt of their 5′ ends. Moreover, 75% of the entire 16,770 *U2* snRNA sequences and 93% of the 208 representative ones in the database contain the GTAG motif in the potential BP-pairing region. Therefore, in humans and likely in many other species as well, a conserved GTAG motif but not a motif evolved to be perfectly complementary to the YTNAY is present in the majority of the *U2* snRNA genes. This non-Watson-Crick-complementarity leads to wobble pairing between U2 and the BP motif, which may result in a less stable helix during the SF3B1-clamping and PRP5-facilitated proof-reading before the A complex formation, providing plasticity for the selection of alternative BPs and/or splice sites. Consistently, BP motif sequences non-complementary to the U2 GTAG motif are more associated with alternative splicing^18,32^. Moreover, different BP locations also regulate alternative splicing^33^. Similarly, in recursive splicing, which requires (skipped) cryptic exons following its 3′AG^34,35^, the suboptimal BP motif and more distant BP, though apparently needed for the zero-nucleotide exon splicing like the 5′ splice sites^36^, may also facilitate the cryptic exon skipping. Therefore, the BP motif sequence and location variations likely contribute to alternative or cryptic splicing, a feature that could also increase transcriptome and proteome diversity. Furthermore, having multi-BPs per 3′ splice site for different tissues or developmental stages may not only suit the more complex cellular context of BP recognition but also help avoid detrimental effects of BP sequence changes on splice site choice for more resilience to genetic variations in evolution as pointed out previously^19^.

### The BP motif variants that cause human diseases

Despite the biological advantages, a disastrous consequence caused by some BP motif variants is human disease. For example, a BP motif variant in the *MSH2* gene causes skipping of exon 16 and results in inherited cancer^37^, or in the *KCNH2* gene causes partial retention of intron 9 and the Long QT syndrome^1,38^. Moreover, mutations in BP *trans*-acting factor genes such as *SF3B1* or *U2* snRNA^3^ drive cancer development^2,3^. Thus, BP motif variations and their recognition by *trans*-acting factors have a critical threshold for proper BP function; beyond which the lariat of critical introns/genes will not form for splicing to occur. However, only 40 pathogenic BP motif variants have been found to cause splicing defects and genetic diseases in the last decades^1^, which is surprisingly low given the exceedingly large number of BPs in the genome. Considering the causative effects of BP dysfunction in diseases, understanding the role of BP motif variants and elucidating their functional impact are essential in dissecting RNA splicing regulation in the physiological and pathophysiological settings. It will also be important for therapeutic applications with small molecules targeting the BP binding proteins such as SF3B1 by spliceostatin A and its analogs or with synthetic lethal genes^39,40^.

### Challenges of prediction, validation, and interpretation of functional BP motifs

Prediction of functional BPs for splicing and experimental validation of BP motif variants involving in disease have been challenging despite the conclusion reported in the original studies. BP prediction software such as BPP, LaBranchoR, or Branchpointer has an AUC (area under the true-versus the false-positive curve) ranging from 0.591 to 0.82 as measured by an independent bioinformatics test^41^. Unexpectedly, when selected Branchpointer-predicted BP motifs were mutated in mini-gene splicing reporters, splicing was not substantially disrupted as assayed by RT-PCR; instead, novel and unpredicted BPs were used as detected by lariat sequencing^42^. A more recent software BPHunter has integrated experimentally identified and computationally predicted BP data, reporting a much higher success rate that approaches 100% and have identified novel BP motif variations^1^. However, the number of tested BPs in that report is small (*n* = 40 BPs) and its false-positive and false-negative rates are not measured.

The imperfect bioinformatics prediction and the lack of most BP information from the exome sequencing approach in detecting BP motif variations are thought to contribute to the surprisingly low rate of pathogenic BP motif variants reported so far^1^. Contributions by alternative BP motifs have not been systematically confirmed in experiments.

In addition to the prediction issues, the indirect BP detection method by mutation/splicing reporter assays of spliced mRNA could easily miss the functional BP at a multi-BP-containing splice site, where different BPs could be used for the same 3′ splice site^19,27^. Here the production of a spliced mRNA only indicates there is at least a functional BP present in the intron, but it does not equate finding the functional BP used for the splicing. Moreover, the mini-gene splicing reporters may not represent the endogenous gene’s BP usage due to the deviation from its native sequence and the chromatin contexts. Additionally, the problem of using different cell lines from the original reports that may have different cell-specific splicing regulators, further complicates the interpretation.

Moreover, the consensus motif of the cryptic (alternative) BP used in *SF3B1* mutant cells is more like the consensus of the insensitive than the canonical BP^33^. The formers contain a higher frequency of purines (YTRAY) while the latter has more enriched C (YTCAY) at the BP-1 positions. How the cryptic BP motifs are chosen and why so many splice sites remain unaffected in the presence of a *SF3B1* mutation in these cells remain elusive.

Furthermore, the molecular mechanisms of the effects of deep intron single nucleotide polymorphism (SNP) variants on human traits or diseases remain to be fully understood. Unraveling the impact of these genetic variants on splicing, especially the BP motifs, would enable us to identify the root causes of these diseases, ultimately facilitating the development of therapeutic interventions.

Lastly, RNA m^6^A modification at splice sites is emerging as a new layer of pre-mRNA splicing regulator. Splice site m^6^A methylation prevents binding of U2AF35/U2AF1 to inhibit RNA splicing^43^. Modification of U2 snRNA and U6 snRNA have also been shown to directly impact mRNA splicing^44,45^. SF3B1 protein expression can be regulated via ubiquitination, and is also phosphorylated by CDK11 during spliceosome transition from the B to B^act^ complex to control pre-mRNA splicing^46,47^. The BP upstream anchoring site and epigenetic factors among others also control or regulate splicing^11,48^. We can speculate that these modifications all could change BP selection to influence splicing, but little is known so far.

## Perspectives about the challenges and issues

**Fig. 2:**
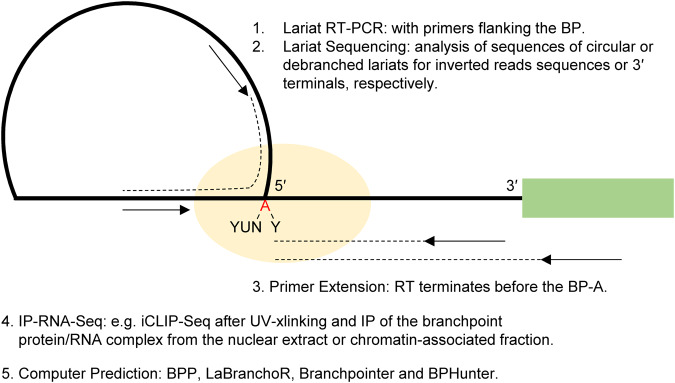
Major methods for direct BP identification or prediction. Each method is briefly described, together with a diagrammed lariat still attached to the downstream exon (green) before the 2^nd^ transesterification. Oval: BP-interacting protein-RNA complex. Arrows: primers, dotted lines: sequence extension in reverse transcription (RT).

Based on the above BP motif variations and related issues, we suggest the following points to consider in studying functional BPs in splicing:

(1) Direct BP detection methods should be used after BP motif prediction (Fig. 2), such as lariat RT-PCR^49^, lariat sequencing^5,21^, primer extension^15^, or RNase protection assay^50^, instead of relying on detecting the spliced RNA alone. The direct detection eliminates false-negative results arising from alternative BP usage when the effects of BP motif variants are examined by spliced RNA only.

(2) Points to consider regarding discrepancies in detecting and validating BPs from transcriptome-wide lariat sequencing: (a) potential differences in multiple cellular contexts used; (b) indirect test of BP usage by using RT-PCR to detect spliced exons; (c) potential differences in BP usage in mini-genes versus endogenous genes; (d) hidden mutations affecting BP usage that may be acquired by the cancer cell during evolving genomic instability. These considerations are particularly important due to the temporal or spatial alternative BP usage in multi-BP introns^19^.

(3) The low rate of pathogenic BPs reported so far perhaps are also attributed by the alternative BP usage for the same exons, besides the prediction/detection issues. This is consistent with the observation that a smaller proportion of pathogenic but not other variants have been identified in the multi-BP than single-BP splice sites^1^. The candidate BPs can be experimentally tested by direct BP detection together with BP mutagenesis/splicing assays. It will be instrumental for identifying candidate BPs by clinical geneticists if an online genome-wide database of annotated alternative BPs of different tissues or developmental stages is available.

(4) The BP motif variations may also help explain how the cryptic BPs are chosen while many unaffected splice sites are also found in splicing factor mutants such as SF3B1 in cancer cells. Some mutated amino acid residues in SF3B1 add negative charges (e.g. the hotspot mutation K700E), causing conformational changes or affecting the interaction with SUGP1^51–54^. This likely causes failure for the HEAT domain to clamp some BP motif-U2 snRNA helixes during PRP5-facilitated proofreading^16^. Particularly affected should be the U2 low-affinity BP motifs, e.g. the CTCAC that is more prevalent at the affected splice sites^33^. These sites are switched in mutants to the cryptic sites that are more enriched with the U2 higher-affinity CTGAC^6,33^. However, the latter high-affinity motifs are generally not used in the wild type; perhaps this was due to the overall weakened 3′ splice sites by a nearby splicing silencer (e.g. G-tracts)^6^, longer distance to the 3′ AG, and/or shorter/weaker Py, besides their deleterious effect of disrupting the mRNA sequences if used. Even more affected should be those 3′ splice sites with both U2 low-affinity motifs and single BPs or those with multi-BP motifs nearby but without the AGEZ^25^. Moreover, for those unaffected splice sites, they may have at least one strong U2-high-affinity BP motif, and those with multi-BPs, the mutant SF3B1 may have chosen alternative, U2 higher-affinity BPs without changing the spliced mRNA. In the latter case, an unaffected splice site in the mutant is not necessarily using the same BP as in the normal cell.

(5) One aspect of the effect of deep intron variants/SNPs on BP usage and cryptic splicing could be through increasing the BP motif base-pairing with the U2 snRNA or interaction with the BP-interacting proteins to enhance cryptic 3′ splice site usage. A consequence could be cryptic exon usage leading to frame shift and non-sense mediated mRNA decay^37^, particularly the cryptic exons of recursive splicing, which is also found in vertebrates including humans^34,55^.

(6) Beyond the above approaches to BP prediction/identification, deep learning methods such as SpliceAI and BigRNA^56,57^, combine more genome as well as transcriptome features including SNP and associated RNA-seq reads to predict variant-associated cryptic or alternative splicing events. These predicted events provide targets with likely splicing consequences for identifying pathogenic BP motif variants facilitated by other software such as BPHunter^1^.

There are still several key questions that remain to be explored. Are there any new *trans*-acting factors or RNA binding proteins (RBP) interacting with BPs yet to be defined? Are there novel disease-relevant BPs yet to be discovered? What will be an effective way to identify any of them? How are the alternative BPs chosen by *trans*-acting factors in cells? How to use specific BPs for therapy without off-target effects by small molecules? With the new developments of CRISPR-based gene editing/screening, now we should be able to test with relative ease the direct involvement of any BP in splicing by mutating the BP motif in the genome or to discover novel RBP factors for alternative/cryptic BP usage.

We hope the insights will help researchers to recognize the significance of accurately identifying and validating functional BPs for the proper interpretation of their roles in RNA splicing. Additionally, we wish to draw attention to the crucial link between BP motif variations and splicing factor mutants (such as SF3B1) in diseases. We believe that this aspect warrants careful consideration by researchers investigating disease pathogenesis associated with these splicing factors, as well as BP biology in general.

### Supplementary information

Reporting-Summary
